# A Probabilistic–Geometric Approach for UAV Detection and Avoidance Systems †

**DOI:** 10.3390/s22239230

**Published:** 2022-11-27

**Authors:** Hae-In Lee, Hyo-Sang Shin, Antonios Tsourdos

**Affiliations:** School of Aerospace, Transport and Manufacturing, Cranfield University, College Road, Cranfield MK43 0AL, UK

**Keywords:** detection and avoidance, Unmanned Aerial Vehicle (UAV), collision probability, differential geometry

## Abstract

This paper proposes a collision avoidance algorithm for the detection and avoidance capabilities of Unmanned Aerial Vehicles (UAVs). The proposed algorithm aims to ensure minimum separation between UAVs and geofencing with multiple no-fly zones, considering the sensor uncertainties. The main idea is to compute the collision probability and to initiate collision avoidance manoeuvres determined by the differential geometry concept. The proposed algorithm is validated by both theoretical and numerical analysis. The results indicate that the proposed algorithm ensures minimum separation, efficiency, and scalability compared with other benchmark algorithms.

## 1. Introduction

There is increasing demand for the development of key technologies for Unmanned Aircraft Traffic Management (UTM), with its versatile applications such as reconnaissance and surveillance, service and support, and logistics in both civilian and military domains. Key aspects in developing safe and efficient UTM services include detection and avoidance systems for multiple Unmanned Aerial Vehicles (UAVs), which is different from the conventional system of Air Traffic Management (ATM) in its scale and diversity of platforms.

The main aim of the detect and avoidance capability in UTM is to guide each UAV to ensure minimum separation with respect to the other UAVs, manned aircrafts, and no-fly zones in the in-flight stage. In contrast to the ATM’s conventional detection and avoidance system, there are several options available for UAV detection and avoidance: rule-based approaches [1,2], geometry-based approaches, artificial potential field algorithms [3,4,5], numerical optimisation methods [6,7,8,9], and learning-based methods [10,11]. Rule-based approaches are easy to implement but usually require different rules depending on the platforms and scenarios and often do not consider simultaneous multi-vehicle avoidance scenarios [12]. Artificial potential field methods are also easy to implement, but these may suffer from the narrow channel problem [13,14]. This occurs when the obstacles are densely located, meaning that the minimum separation is not guaranteed near the local minima. Numerical optimisation methods can guarantee the minimum separation as well as optimising the energy or time, but the computational load is higher than the rule-based or artificial potential field methods [15].

One issue of the aforementioned detection and avoidance methods is that most of them are developed under the assumption that the obstacles have circular or elliptical shapes [16]. This assumption may not be practical or efficient considering that no-fly zones are usually declared to be large in scale and as 4D polygons. Approximating a large polygonal zone as a cylindrical shape can lead to unnecessary deviation from the original flight plan, reducing the overall efficiency, and potentially risking mission failure. In urban environments, there could be even no feasible path between the densely located buildings that are approximated as cylindrical shapes. Hence, the consideration of irregularly shaped obstacles in tactical de-confliction is a key element of a UTM solution expanding the operational boundary to challenging environments.

Another issue to be considered is the uncertainties in the UAVs’ relative position to the moving intruders. There have been several methods proposed to quantify the collision risk as collision probability considering these sensor uncertainties: the integration of a multi-variate Gaussian probability density function [17,18,19], Gaussian mixture [20,21], and Monte-Carlo simulations [22,23,24]. The main discussions have been focused on how to precisely and computationally effectively quantify the collision probability by selecting appropriate error distribution models and approximating their integration. However, how the computed collision probability can be effectively used in the collision avoidance manoeuvre remains largely unknown.

This paper proposes a probabilistic–geometric collision avoidance algorithm that can consider multiple irregularly shaped no-fly zones and sensor uncertainties of multiple intruder UAVs. The collision probability is computed by integrating a multi-variate Gaussian probability density function [17] and then is used to compute the desired avoidance manoeuvre. Here, the avoidance manoeuvre is computed by the differential geometry concept [25,26,27] to analytically derive the conditions to guarantee the minimum separation. A key principle is to identify the conflict on the line-of-sights to the waypoint and compute the heading angle rate to guarantee avoidance while minimising the control effort. The performance metrics are set as three main aspects—safety, scalability, and efficiency—and the validity of the proposed algorithm is shown around these metrics in numerical simulations. The results are compared with two commonly used collision avoidance algorithms: artificial potential field [5] and particle swarm optimisation [9]. Combining the collision probability and differential geometry concept, the proposed approach can guarantee the safety and efficiency comparable to optimisation-based methods, while retaining the scalability similar to the artificial potential field. Note that the authors’ previous work [28] presented initial results using the differential geometry concept but did not contain the collision probability considering the sensor uncertainties and only considered a single moving intruder UAV. In addition, the parameter analysis with respect to different scenarios is presented in this work for the rigorous validation of safety and scalability. Hence, the contribution of this paper can be summarised as follows:

- The proposition of a new collision detection and avoidance algorithm that achieves the following properties:
  - Multiple irregularly shaped obstacles and moving intruders can be considered in tactical de-confliction;
  - Uncertainties in UAVs’ relative position are considered in determining the collision avoidance manoeuvre by utilising the collision probability;
  - Minimum separation for safety can be analytically proven by differential geometry concept.
- Validation of performance of the proposed algorithm using analytical and numerical analysis.
- Demonstration of the safety, scalability, and efficiency of the proposed approach in comparison with other well-known benchmark algorithms.

The rest of the paper is composed as follows: the problem formulation and some definitions are given in Section 2. In Section 3, the proposed collision avoidance algorithm and its theoretic analysis is addressed. The numerical simulations in Section 4 validate and verify the proposed algorithm. Conclusions are given in Section 5.

## 2. Problem Formulation

Consider a 2D scenario with a UAV guided to a waypoint, and there exist multiple intruder UAVs and polygonal obstacles. There exist position errors especially with respect to the intruder UAVs, and we assume the following conditions:

- The relative position error with the intruder UAVs satisfies the Gaussian distribution. This assumption was validated by the empirical studies and used in many related works [19].
- There exists no correlation between the estimated positions of UAVs. In the scenarios considered in this paper, it is assumed that the UAV estimates the positions of intruders and its position using onboard sensors. Under these scenarios, it is reasonable to assume that the estimated positions of UAVs are uncorrelated. This assumption makes the covariance matrix of the relative position estimates the summation of individual UAVs’ covariance matrices [17].
- The ground speeds of the vehicle and intruders are assumed to be constant at *V* and $V_{in_{i}}$, respectively.

**Figure 1:**
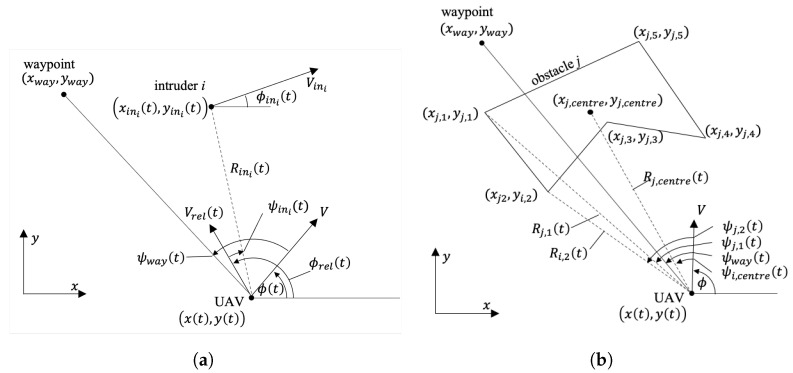
Relative geometries of a UAV. (**a**) Relative geometry to a moving intruder. (**b**) Relative geometry to a polygonal obstacle.

Then, the relative geometry of the UAV to the *i*-th intruder vehicle is shown in Figure 1a. It is assumed that the position $\left(x_{in_{i}}\left(t\right),y_{in_{i}}\left(t\right)\right)$, ground speed $V_{in_{i}}$, and heading angle $\phi_{in_{i}}\left(t\right)$ of the intruder are known within a certain range, either because the intruder is cooperative or because its position and velocity are measured through sensors. Then, the relative velocity $V_{rel}\left(t\right)$ and its direction $\phi_{rel}\left(t\right)$ can be computed. The range $R_{in_{i}}\left(t\right)$ and bearing angle $\psi_{in_{i}}\left(t\right)$ of the intruders are computed with respect to the position and *relative* velocity of the UAV.

The relative geometry of the UAV to the *j*-th polygonal obstacle is shown in Figure 1b. It is assumed that the polygonal obstacles include no-fly zones, buildings, and other obstacles that are fixed for a given time span, and the position of their *k*-th feature points, $\left(x_{j,k}\left(t\right),y_{j,k}\left(t\right)\right)$ for all *k*, is known. The range $R_{j,k}\left(t\right)$ and bearing $\psi_{j,k}\left(t\right)$ of the *j*-th obstacle’s *k*-th feature points are computed with respect to the position and velocity of the UAV, respectively. For instance, $R_{j,1}\left(t\right)$ and $R_{j,2}\left(t\right)$ are plotted in Figure 1b as the range with respect to $(x_{j,1},y_{j,1}$ and $\left(x_{j,2},y_{j,2}\right)$ respectively, and they are defined the same for the other feature points, $\left(x_{j,k}\left(t\right),y_{j,k}\left(t\right)\right)$ for k∈[3,5]. The range $R_{j,centre}\left(t\right)$ and bearing $\psi_{j,centre}\left(t\right)$ of the *j*-th obstacle’s centre are defined to distinguish the feature points from other obstacles. The range of the whole *j*-th obstacle, $R_{j}\left(t\right)$, is defined as the minimum distance of the UAV to each nodes and edges of the obstacle. For instance, as the closest point of the obstacle *j* to the UAV in Figure 1b is the fourth feature point, $R_{j}\left(t\right)$ would be the same as $R_{j,4}\left(t\right)$ in this case. If a certain edge is the closest to the UAV, $R_{j}\left(t\right)$ is the distance to the nearest point of the edge.

Based on the relative geometries of the UAV, the recognition and collision is defined as in [27]:

**Definition** **1** (Recognition). *The UAV is able to recognise*

- *the intruder i if $R_{in_{i}}\left(t\right)<R_{RC,i}$;*
- *the polygonal obstacle j if $R_{j}\left(t\right)<R_{RC,j}$.*

*where $R_{RC,i}$ and $R_{RC,j}$ are the recognition range of the i-th intruder and the j-th obstacle, respectively.*

**Definition** **2** (Collision). *The UAV collides with*

- *the intruder i if $R_{in_{i}}\left(t\right)<R_{0}$,*
- *the polygonal obstacle j if $R_{j}\left(t\right)<R_{0}$;*

*where $R_{0}$ is the minimum separation.*

## 3. Collision Avoidance Algorithm

### 3.1. Collision Probability Computation

The collision probability is defined as the probability that the UAV would collide if maintaining the current heading angle. This can be calculated by integrating the probability density function as [17]

(1) $$P\left(t\right)={\;\int \int \;}_{x^{2}+y^{2}\leq R_{0}^{2}}f\left(x,y\right)\;dx\;dy,$$

where f(x,y) is the probability density function of the relative position error of the intruder UAV. Please note that the relative *x*–*y* position of the UAV varies with time *t* but is denoted as *x* and *y* for simplicity in integration. Assuming the Gaussian distribution with mean $\left(\mu_{x},\mu_{y}\right)$ and standard deviation $\left(\sigma_{x},\sigma_{y}\right)$, it can be defined as

(2) $$f\left(x,y\right)=\frac{1}{2\pi \sigma_{x}\sigma_{y}}\exp\left[-\frac{1}{2}\left(\frac{{\left(x-\mu_{x}\right)}^{2}}{\sigma_{x}^{2}}+\frac{{\left(y-\mu_{y}\right)}^{2}}{\sigma_{y}^{2}}\right)\right].$$

Converting the Cartesian coordinate to the polar coordinate, i.e., $x=\sqrt{\frac{\sigma_{x}}{\sigma_{y}}}r\cos\theta$, $y=\sqrt{\frac{\sigma_{y}}{\sigma_{x}}}r\sin\theta$, $\mu_{x}=\sqrt{\frac{\sigma_{x}}{\sigma_{y}}}\rho \cos\theta_{\rho }$, and $\mu_{y}=\sqrt{\frac{\sigma_{y}}{\sigma_{x}}}\rho \sin\theta_{\rho }$, the collision probability is computed as

(3) $$P\left(t\right)=\frac{1}{2\pi \sigma_{x}\sigma_{y}}\int_{0}^{R_{0}}r\exp\left(-\frac{r^{2}+\rho^{2}}{2\sigma_{x}\sigma_{y}}\right)\int_{0}^{2\pi }\exp\left(\frac{r\rho \cos(\theta -\theta_{\rho })}{\sigma_{x}\sigma_{y}}\right)\;d\theta \;dr,$$

where μ, ρ, θ, and $\theta_{\rho }$ are also functions of time *t* but denoted for simplicity as integration variables.

Here, the mean distance between the UAVs ρ is computed at the closest approach point as

(4) $$\rho =\left\{\begin{array}{ccc} R_{in_{i}}\left(t\right)\left|\sin(\psi_{in_{i}}\left(t\right)-\phi_{rel_{i}}\left(t\right))\right|, & \; & \;\mathrm{if}\;|\psi_{in_{i}}\left(t\right)-\phi_{rel_{i}}\left(t\right)|<\frac{\pi }{2}\\ R_{in_{i}}\left(t\right), & \; & \;\mathrm{otherwise}. \end{array}\right.$$

### 3.2. Conflict Detection Method

Any conflicting intruders or obstacles are detected considering the computed collision probability. For the recognised intruder *i*, the bearing angle ensuring the minimum separation is obtained as

(5) $$\begin{array}{cc} \psi_{in_{i},L}\left(t\right) & =W\left(\psi_{in_{i}}\left(t\right)+\sin^{-1}\frac{R_{p}\left(t\right)}{R_{in_{i}}\left(t\right)}\right),\\ \psi_{in_{i},R}\left(t\right) & =W\left(\psi_{in_{i}}\left(t\right)-\sin^{-1}\frac{R_{p}\left(t\right)}{R_{in_{i}}\left(t\right)}\right). \end{array}$$

where W(·) wraps the angle to [−π,π], and the subscripts *L* and *R* stand for the left and right-hand side with respect to the line-of-sight to the *i*-th intruder. Here, $R_{p}$ is introduced to ensure minimum separation under the existence of uncertainties, using the computed collision probability as

(6) $$R_{p}\left(t\right)=R_{0}+3\sqrt{\sigma_{x}\sigma_{y}}P\left(t\right).$$

The physical meaning behind $R_{p}\left(t\right)$ is that it enlarges the avoidance manoeuvre approximately up to the 3−σ line when the collision probability is high, whereas if the probability is low, the minimum separation $R_{0}\left(t\right)$ is introduced. This ensures the avoidance of the moving uncertain intruder *i* with minimal detour.

In a similar manner for each recognised polygonal obstacle *j*, two nodes that are most at risk of collision can be identified from

(7) $$\begin{array}{cc} k_{L} & =\arg\max_{k}W\left(\psi_{j,k}\left(t\right)+\sin^{-1}\frac{R_{0}}{R_{j,k}\left(t\right)}-\psi_{j,centre}\left(t\right)\right),\\ k_{R} & =\arg\min_{k}W\left(\psi_{j,k}\left(t\right)-\sin^{-1}\frac{R_{0}}{R_{j,k}\left(t\right)}-\psi_{j,centre}\left(t\right)\right), \end{array}$$

where the subscripts *L* and *R* stand forthe left and right-hand side with respect to the line-of-sight to the obstacle’s centre.

The bearing angle of the two nodes to ensure the minimum separation is

(8) $$\begin{array}{cc} \psi_{j,L}\left(t\right) & =W\left(\psi_{j,k_{L}}\left(t\right)+\sin^{-1}\frac{R_{0}}{R_{j,k_{L}}\left(t\right)}\right),\\ \psi_{j,R}\left(t\right) & =W\left(\psi_{j,k_{R}}\left(t\right)-\sin^{-1}\frac{R_{0}}{R_{j,k_{R}}\left(t\right)}\right). \end{array}$$

Then, the union of the conflicting intervals can be obtained as

(9) $$I=\left(\underset{i}{⋃}\left[\psi_{in_{i},R}\left(t\right),\psi_{in_{i},L}\left(t\right)\right]\right)\cup \left(\underset{j}{⋃}\left[\psi_{j,R}\left(t\right),\psi_{j,L}\left(t\right)\right]\right).$$

**Figure 2:**
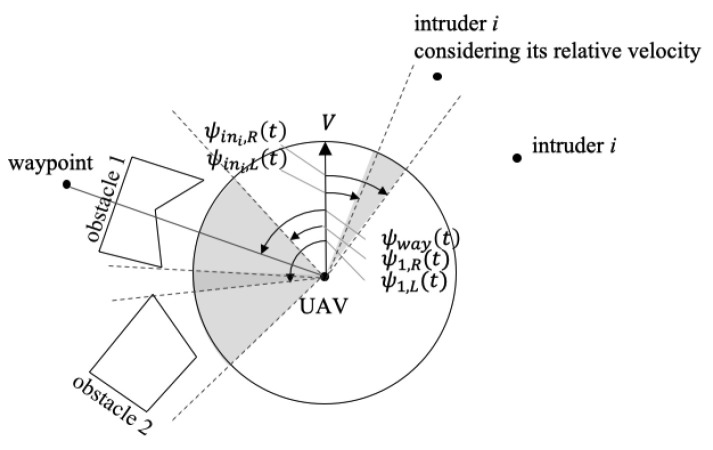
Conflict detection and resolution intervals.

This set of intervals shows which line-of-sight leads to potential collision, as shown in Figure 2. Since the desired line-of-sight heads to the waypoint, the conflict is detected if

(10) $$\psi_{way}\left(t\right)\in I.$$

### 3.3. Conflict Resolution Method

If the conflict is detected, let us define the largest interval $[\psi_{R}\left(t\right),\psi_{L}\left(t\right)]\subset I$ which contains $\psi_{way}\left(t\right)$. The conflict can be resolved by steering the UAV’s heading angle either by $\psi_{R}\left(t\right)$ or $\psi_{L}\left(t\right)$.

Note that this interval proposed in this work is different from the union of the intervals that contain $\psi_{way}\left(t\right)$, i.e.,

(11) $$\begin{array}{cc} I^{\prime }= & \left(\underset{i}{⋃}\left\{\left.\left[\psi_{in_{i},R}\left(t\right),\psi_{in_{i},L}\left(t\right)\right]\right|\psi_{way}\left(t\right)\in \left[\psi_{in_{i},R}\left(t\right),\psi_{in_{i},L}\left(t\right)\right]\right\}\right)\\ \; & \cup \left(\underset{j}{⋃}\left\{\left.\left[\psi_{j,R}\left(t\right),\psi_{j,L}\left(t\right)\right]\right|\psi_{way}\left(t\right)\in \left[\psi_{j,R}\left(t\right),\psi_{j,L}\left(t\right)\right]\right\}\right) \end{array}$$

which has been commonly used in previous works on differential geometry based collision avoidance [25,27]. The physical meaning of computing I and deriving the interval $\left[\psi_{R}\left(t\right),\psi_{L}\left(t\right)\right]$ is to include the obstacle/intruder that does not directly intersect the UAV’s line-of-sight to the waypoint but overlaps with another in direct conflict. This reduces the UAV’s detour from the waypoint by foreseeing the potential conflicts and resolves the chattering problem mentioned in the previous works. For instance, the set of intervals I is visualised as a grey area in Figure 2. Two intervals against the fixed obstacles 1 and 2 are plotted with respect to their line-of-sight, and one interval against the moving intruder *i* is plotted considering its relative velocity. The largest interval in I containing the waypoint is $\left[\psi_{2,L}\left(t\right),\psi_{1,R}\left(t\right)\right]$, whereas the interval obtained from $I^{\prime }$ is $\left[\psi_{1,L}\left(t\right),\psi_{1,R}\left(t\right)\right]$. Steering of the UAV towards $\psi_{1,L}\left(t\right)$ may result in an unnecessary detour or chattering issue.

Once the interval $\left[\psi_{R}\left(t\right),\psi_{L}\left(t\right)\right]$ is obtained from I, the desired heading angle change is determined to minimise the detour from the waypoint as

(12) $$\psi_{d}\left(t\right)=\left\{\begin{array}{ccc} \psi_{R}\left(t\right), & \; & \;\mathrm{if}\;|\psi_{R}\left(t\right)-\psi_{way}\left(t\right)|<|\psi_{L}\left(t\right)-\psi_{way}\left(t\right)|;\\ \psi_{L}\left(t\right), & \; & \;\mathrm{otherwise}. \end{array}\right.$$

This choice of the heading angle change is made to minimise the time to reach the waypoint. Otherwise, to reduce control efforts, one may consider choosing the heading angle as

(13) $$\psi_{d}\left(t\right)=\left\{\begin{array}{ccc} \psi_{R}\left(t\right), & \; & \;\mathrm{if}\;|\psi_{R}\left(t\right)|<|\psi_{L}\left(t\right)|;\\ \psi_{L}\left(t\right), & \; & \;\mathrm{otherwise}. \end{array}\right.$$

The heading angle control to achieve the desired change $\psi_{d}$ is then suggested as

(14) $$\dot{\phi }\left(t\right)=\frac{V_{d}\left(t\right)}{\sqrt{R_{d}^{2}\left(t\right)-R_{0}^{2}}}\mathrm{sgn}\psi_{d}\left(t\right)+K\psi_{d}\left(t\right),$$

where $V_{d}\left(t\right)$ and $R_{d}\left(t\right)$ are the relative velocity and range of the intruder and obstacle at the line-of-sight of $\psi_{d}\left(t\right)$, respectively, and K>0 is the control gain of the heading angle.

### 3.4. Minimum Separation Analysis

The summary of the proposed algorithm is shown in Algorithm 1. This algorithm enables the consideration of multiple irregularly shaped obstacles and moving intruders by measuring their current relative positions and velocities only. Lines 3–5 enable the consideration of sensor uncertainties in the avoidance manoeuvre, and Line 18 is critical in analytically proving the minimum separation. It is shown that the computations are mainly divided into iterative loops for each moving intruder and fixed polygonal obstacle, which implies that the computational complexity of the proposed algorithm is linearly increasing.

**Algorithm 1** Collision Avoidance Algorithm**Input:**$\psi_{way}\left(t\right)$,$\psi_{in_{i}}\left(t\right)$,$\psi_{j,k}\left(t\right)$,$\psi_{j,centre}\left(t\right)$,ϕ(t),$\phi_{in_{i}}\left(t\right)$,$R_{in_{i}}\left(t\right)$,$R_{j}\left(t\right)$.**Output:**$\dot{\phi }\left(t\right)$.  1: **for** each moving intruder UAV *i* **do**      12:         $\left[\psi_{j,R},\psi_{j,L}\right]$← Equation (8);  2:     **if** $R_{in_{i}}\left(t\right)<R_{RC,i}$ **then**           13:     **end if**  3:         ρ(t)←Equation (4);           14: **end for**  4:         P(t)←Equation (3);          15: I←Equation (10);  5:         $R_{p}\left(t\right)$←Equation (6);          16: **if**
$\psi_{way}\left(t\right)\in I$
**then**  6:         $[\psi_{in_{i},R}\left(t\right),\psi_{in_{i},L}\left(t\right)]\leftarrow$Equation (5);        17:     $\psi_{d}\left(t\right)$←Equation (12);  7:     **end if**                      18:     $\dot{\phi }\left(t\right)$←Equation (14);  8: **end for**                    19: **else**  9: **for** each fixed obstacle *j* **do**         20:     $\dot{\phi }\left(t\right)\leftarrow K\left(\psi_{way}\left(t\right)-\phi \left(t\right)\right)$10:     **if** $R_{j}\left(t\right)<R_{RC,j}$ **then**                 21: **end if**11:         $\left[k_{R},k_{L}\right]$←Equation (7);         22: **return**
$\dot{\phi }\left(t\right)$

If the desired heading angle change $\psi_{d}\left(t\right)$ is properly computed, it has been proven that the minimum separation is guaranteed through the suggested heading angle control [25,27]. Its characteristics and proof are briefly addressed in the following theorems:

**Theorem** **1.** *If the ground speed of the main UAV is greater than or equal to that of the intruder UAV, i.e., $V\geq V_{in_{i}}$, the proposed collision avoidance guarantees the minimum separation.*

**Proof.**  The guarantee of the minimum separation is identical to the convergence of the desired heading angle change to zero. If there exists a non-zero interval $\left[\psi_{R}\left(t\right),\psi_{L}\left(t\right)\right]$, it means that the UAV is still in conflict, and thus the desired heading angle change, $\psi_{d}\left(t\right)$, is also non-zero. Hence, this proves that the minimum separation guarantee can be converted to showing a Lyapunov stability of $\psi_{d}\left(t\right)$, with the Lyapunov function as

(15) $$V\left(\psi_{d}\left(t\right)\right)=\frac{1}{2}\psi_{d}^{2}\left(t\right).$$

As it is clear that V(0)=0, and there exists a positive value *a* satisfying $V\left(\psi_{d}\left(t\right)\right)\geq a||\psi_{d}\left(t\right)\left|\right|$, the suggested function is a valid Lyapunov function [29]. Substituting Equation (14) into ${\dot{\psi }}_{d}\left(t\right)={\dot{\phi }}_{rel}\left(t\right)-\dot{\phi }\left(t\right)$ for the obstacle/intruder in conflict, the time derivative of the Lyapunov function is computed as

(16) $$\begin{array}{cc} \dot{V}\left(\psi_{d}\left(t\right)\right) & =\psi_{d}\left(t\right)\left({\dot{\phi }}_{rel}\left(t\right)-\left(\frac{V_{d}\left(t\right)}{\sqrt{R_{d}^{2}\left(t\right)-R_{0}^{2}}}\mathrm{sgn}\psi_{d}\left(t\right)+K\psi_{d}\left(t\right)\right)\right)\\ \; & =-K\psi_{d}^{2}\left(t\right)-\left(\frac{V_{d}\left(t\right)}{\sqrt{R_{d}^{2}\left(t\right)-R_{0}^{2}}}\mathrm{sgn}\psi_{d}\left(t\right)-{\dot{\phi }}_{rel}\left(t\right)\right)\psi_{d}\left(t\right). \end{array}$$

Considering that the obstacles are assumed to be fixed and that intruder isnon-manoeuvring, $\mathrm{sgn}{\dot{\phi }}_{rel}\left(t\right)=\mathrm{sgn}\psi_{d}\left(t\right)$. In addition, the assumption on $V\geq V_{in_{i}}$ gives $|{\dot{\phi }}_{rel}\left(t\right)|\leq V_{d}\left(t\right)/\sqrt{R_{d}^{2}\left(t\right)-R_{0}^{2}}$ for all fixed obstacles and moving intruders. Hence, from Equation (16), the Lyapunov function satisfies

(17) $$\dot{V}\left(\psi_{d}\left(t\right)\right)\leq -K\psi_{d}^{2}\left(t\right)<0,\;\forall \psi_{d}\left(t\right)\neq 0.$$

This proves that the desired heading angle change asymptotically converges to 0, guiding the UAV on the line-of-sight to avoid the collision. □

**Theorem** **2.** *For the ground speed of the main UAV less than that of the intruder UAV, i.e., $V<V_{in_{i}}$, the proposed collision avoidance guarantees the minimum separation if*

(18) $$|\phi_{in_{i}}\left(t\right)-\phi \left(t\right)-\psi_{d}\left(t\right)|\leq \sin^{-1}\frac{V}{V_{in_{i}}}$$

**Figure 3:**
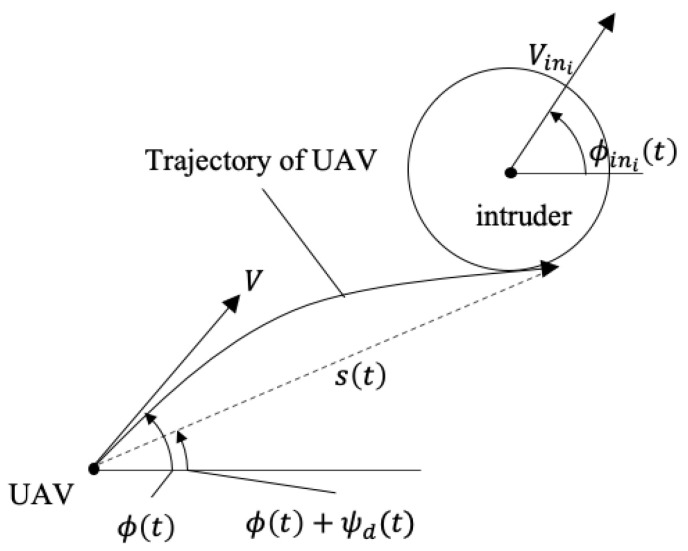
Collision avoidance geometry of a UAV to an intruder.

**Proof.**  From the geometric relationship in Figure 3, the collision avoidance trajectory of the UAV with respect to a moving intruder satisfies

(19) $$\frac{s(t)}{t_{c}}=V_{in_{i}}\cos\left(\phi_{in_{i}}\left(t\right)-\phi \left(t\right)-\psi_{d}\left(t\right)\right)-\sqrt{V^{2}-V_{in_{i}}^{2}\sin^{2}\left(\phi_{in_{i}}\left(t\right)-\phi \left(t\right)-\psi_{d}\left(t\right)\right)},$$

where s(t) is the length of the tangent line to avoid the collision, and $t_{c}$ is the time for the UAV to reach the point of tangency. If $V^{2}-V_{in_{i}}^{2}\sin^{2}\left(\phi_{in_{i}}\left(t\right)-\phi \left(t\right)-\psi_{d}\left(t\right)\right)\geq 0$, there exists a feasible trajectory s(t), and hence the UAV is able to avoid the intruder. For the details of the proof, refer to [25]. □

**Theorem** **3.** *For $V\geq V_{in_{i}}$ and the maximum heading angle rate limited by $r_{max}$, the minimum separation is guaranteed if*

(20) $$R_{d}\left(t\right)\geq V_{d}\left(t\right)\frac{\psi_{d}\left(t\right)}{r_{max}}+R_{0},$$

*where $V_{d}\left(t\right)$ and $R_{d}\left(t\right)$ are the relative velocity and range of the obstacle/intruder at the line-of-sight of $\psi_{d}\left(t\right)$, respectively.*

**Proof.**  The minimum time required to complete the avoiding turn is

(21) $$t_{c}\geq \frac{\psi_{d}\left(t\right)}{r_{max}}.$$

In order to complete the turn before colliding, the distance of the UAV and obstacle/intruder should satisfy

(22) $$R_{d}\left(t\right)-R_{0}\geq V_{d}\left(t\right)t_{c}\geq V_{d}\left(t\right)\frac{\psi_{d}\left(t\right)}{r_{max}}.$$

□

## 4. Numerical Simulations

### 4.1. Simulation Setup

**Figure 4:**
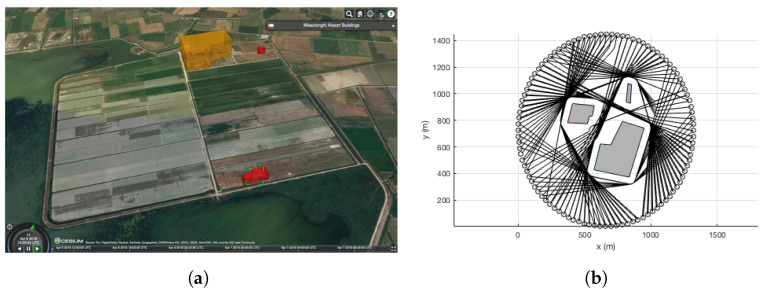
Simulation scenario. (**a**) Test site, Messolonghi Airport in Greece. (**b**) Simulation environment.

Numerical simulations are conducted to validate the performance of the proposed collision avoidance algorithm. Three fixed obstacles are modelled from the no-fly zones and buildings near the test site, Messolonghi Airport in Greece as shown in Figure 4a, but the distances between the obstacles are set denser than the actual environment, creating a more challenging environment for rigorous validation. The simulation environment and sample trajectories around the obstacles are shown in Figure 4b. The velocity of the UAVs, *V* and $V_{in_{i}}$’s are set as 14 m/s, and the desired minimum separation $R_{0}$ is 50 m. Sensor noise is added to the intruder vehicles only, with $\sigma_{x}=\sigma_{y}=\sigma =50$ m. A simple first-order low-pass filter is implemented with a time constant τ=0.01. For rigorous validation, 100 different trajectories of an intruder UAV are set uniformly around the scenario, with the starting and end points opposite to each other in a circle with the radius of 693 m, which is defined in consideration of obstacles. In order to create the intruder UAV’s trajectories as not colliding with the fixed obstacles, the proposed differential geometry concept is used for the fixed obstacles only.

The performance of the proposed collision avoidance algorithm is evaluated in three main metrics: minimum separation distance, total flight time, and computation time. These metrics are selected to verify the safety, efficiency, and scalability of the algorithm, respectively. In addition, these metrics of the proposed method—noted as the differential geometry concept (DGC)—are compared with two benchmark algorithms: artificial potential field (APF) and particle swarm optimisation (PSO). APF was set using repulsive fields against the intruders and fixed obstacles and an attractive field to reach the waypoint as

(23) $$\dot{\phi }\left(t\right)=k_{way}\psi_{way}\left(t\right)\left(e^{-\frac{R_{way}\left(t\right)}{R_{0}}}+1\right)-\sum_{i}k_{in}\psi_{in_{i}}\left(t\right)e^{-\frac{R_{in_{i}}\left(t\right)}{R_{0}}}-\sum_{j}k_{obs}\psi_{j,centre}\left(t\right)e^{-\frac{R_{j,centre}\left(t\right)-r_{j}}{R_{0}}},$$

where the gains $k_{way}$, $k_{in}$, and $k_{obs}$ are design parameters to adjust the repulsive and attractive forces, and $r_{j}$ is the radius of the *j*-th obstacle. Note that conventional APF can consider only circular/ellipsoidal obstacles. PSO is set to minimise the following cost function:

(24) $$\min J\left(x,y\right)=t_{f}+\lambda f\left(x,y\right)+\lambda \sum_{i}g_{i}\left(x,y\right),$$

where $f\left(x,y\right)=-\min\left(1-\frac{R_{way}\left(t_{f}\right)}{10},0\right)$, and $g_{i}\left(x,y\right)=\int_{0}^{t_{f}}\max\left(1-\frac{R_{j}\left(t\right)}{R_{0}},0\right)dt$. The parameter λ is introduced to impose the constraint. The design parameter settings for DGC, APF, and PSO are summarised in Table 1. Note that h(·) is a step function to impose the gains for certain ranges only.

### 4.2. Simulation Results for a Single Scenario

**Figure 5:**
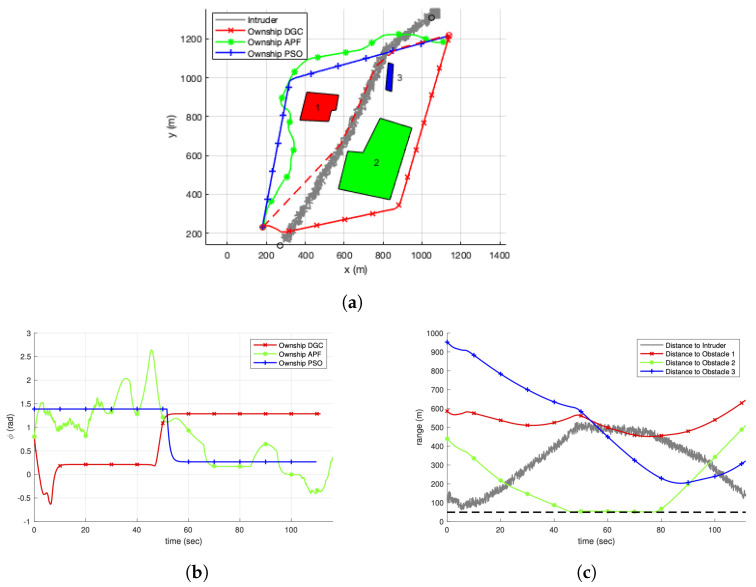
Single scenario comparison. (**a**) Trajectory. (**b**) Flight angle. (**c**) Distance to obstacles and intruder.

Simulation results are presented for a single scenario to intuitively show how the selected algorithms perform. For a single scenario, the trajectories of the selected algorithms are shown in Figure 5a. The intruder vehicle goes through the obstacles to reach the waypoint with its sensor uncertainties, and the trajectory of the main UAV without considering the intruder, shown with a dashed line, also goes through the obstacles, resulting in the inevitable collision with the intruder. The UAV with DGC, APF, and PSO instead detours the obstacles to avoid the collision with the intruder. The inefficient behaviour of APF is caused by the fact that the obstacles are considered as circular shapes, and their repulsive forces are summed in a dense environment. DGC shows a right turn at the initial phase, while PSO shows a left turn. Their efficiency is clearly compared in Figure 5b, where the heading angles of the trajectories are shown with respect to the simulation time. Comparing the end time of the simulation, PSO shows the shortest simulation time, and DGC is the second efficient algorithm. The efficiency of PSO is caused by its nature knowing the trajectory of the intruder in advance. Considering that DGC can be applied for uncooperative intruders where their future flight plans are not shared, this implies that the proposed algorithm can efficiently reach the target point with a reasonable flight time, while maintaining the safety to guarantee the minimum separation. The distance between the UAV with DGC and other obstacles is shown in Figure 5c. Each solid line shows the distance from the UAV to the obstacles during the simulation, and the dashed line is the minimum safety distance of 50 m. This shows that the minimum separation is always guaranteed to all obstacles despite the uncertainties in the intruder’s measurements.

### 4.3. Simulation Results for Monte-Carlo Simulations

**Figure 6:**
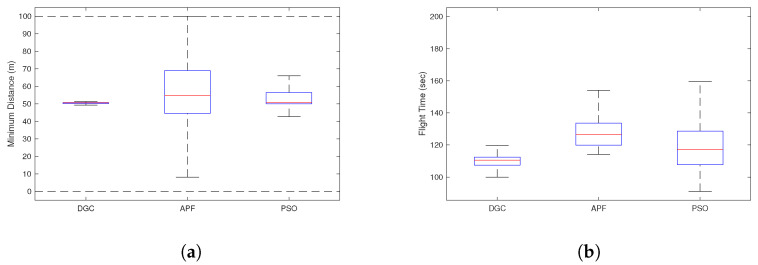
Monte-Carlo simulation comparison. (**a**) Minimum distance. (**b**) Total flight time.

For rigorous validation, Monte-Carlo simulations are conducted with 100 different scenarios. For each algorithm, the minimum separation distance and total flight time are shown in Figure 6. In Figure 6a, it is shown that DGC strictly guarantees the minimum separation, 50 m, in almost all scenarios, whereas the minimum distance of PSO shows large variance and is sometimes not guaranteed, and the variance is much larger with APF. Note that the negative distance means that the UAV is inside the polygonal obstacle. This shows that APF suffers from a widely known issue—a narrow channel problem—in challenging environments. In Figure 6b, DGC shows the shortest total flight time, which is directly linked to its efficiency. PSO has better efficiency for some cases, but both its mean and variance of the total flight time are longer than DGC. Note that PSO used in this paper is computed with the future trajectory of the intruder UAV, which contributes to improving the efficiency. Although the proposed DGC is computed with the current position and velocity of the intruder UAV, it achieves better efficiency than PSO in an average sense. The flight time of APF is much longer than the other algorithms for a larger detour, as inferred from Figure 6a. Another important performance metric to be compared is the computational cost, and the average computational time for each scenario was 0.2 s, 0.1 s, and 4.6 s for DGC, APF, and PSO, respectively for MATLAB operating with a 2.8 GHz Intel i5.

The simulation results are summarised in Table 2. The ranged values represent lower and upper adjacent values, respectively. Comparing the three performance metrics—minimum distance, flight time, and computational cost—the proposed algorithm DGC guarantees safety with much higher efficiency than APF and is much more scalable than PSO by fully utilising its computation of collision probability and analytical guarantee of differential geometry concept.

## 5. Parameter Analysis

### 5.1. Effect of Sensor Noise Variance

For rigorous validation, the numerical simulations are repeated for different variances of sensor noise. The scenario is set in the same way as in Figure 5a, but different sensor noises with a standard deviation of σ∈[0,100] m are applied. The time constant of the low-pass filter should vary, and it is designed according to the following equation:

(25) $$\tau =\frac{1}{\Delta t}\max\left(0.01,0.002\sigma \right),$$

where Δt is the sample time, which is set as 0.1 s.

**Figure 7:**
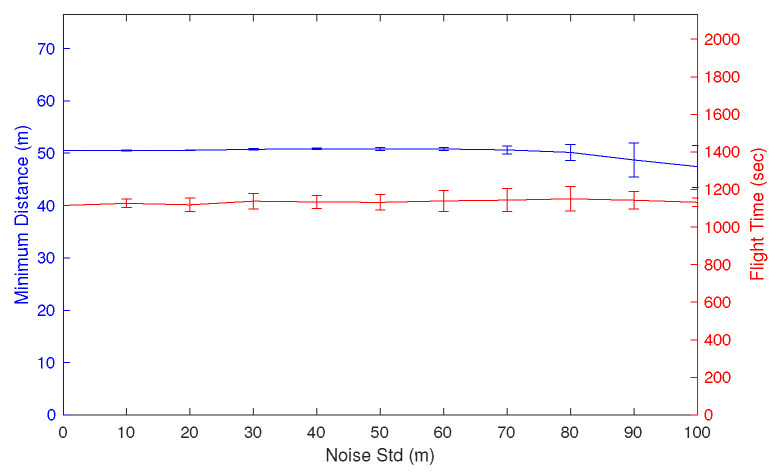
Effect of sensor noise variance on the minimum distance and total flight time.

The mean and variance of resultant minimum distance and the flight time are shown in Figure 7, where the simulations are repeated 100 times for each case. It is shown from the blue line that the minimum distance of 50 m is well-guaranteed with a standard deviation less than 80 m, which is sufficient considering the scenario scale. In addition, although the sensor noise increases, the red line shows that the detour of the UAV remains minimal.

### 5.2. Effect of the Number of Intruders

**Figure 8:**
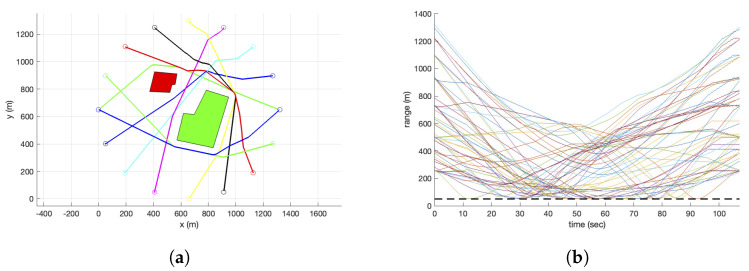
Collision avoidance with 9 UAVs. (**a**) Trajectories. (**b**) Distance to each intruder.

The effect of the number of intruders on the detection and avoidance performance is analysed to investigate the scalability of the proposed algorithm. For instance, the trajectories of 9 UAVs successfully avoiding the collision with all the other UAVs and no-fly zones are shown in Figure 8a. Here, the UAVs are trying to reach the opposite point of the circle around the fixed obstacles, starting at the same time. The distance plot in Figure 8b shows 36 distances between the 9 UAVs along the flight time. Note that each solid line represents the distance between two UAVs, and as there are 36 pairs of distance for 9 UAVs, the legends are omitted. However, it is shown that all the solid lines are above the dashed line, which is the minimum separation. Hence, it is always guaranteed to be above the minimum separation.

**Figure 9:**
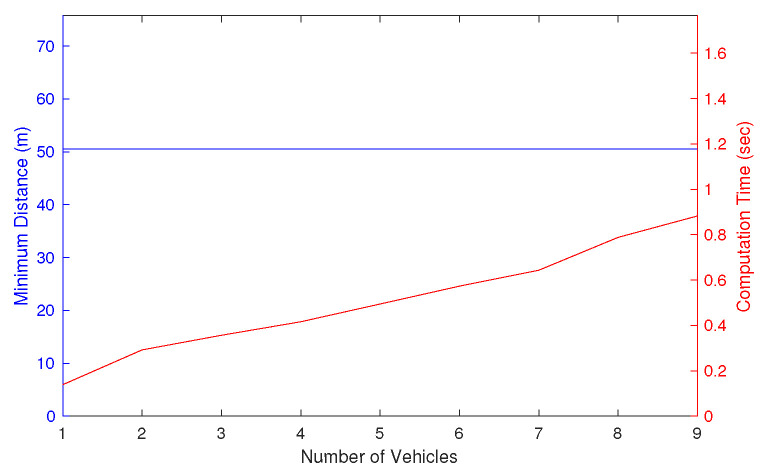
Effect of the number of intruders on the minimum distance and computation time.

By increasing the number of UAVs from 1 to 9, the separation distance is guaranteed above the threshold, as shown in Figure 9. Note that it is clear that the proposed algorithm cannot always guarantee the minimum separation if the simulation environment is challenging, i.e., when there is no feasible solution. While guaranteeing the safety, the computational complexity increases linearly with respect to the number of UAVs, as expected from the analysis. Considering that some cooperative collision avoidance or multi-UAV trajectory optimisations show NP-hardness [30], this implies the scalability of the proposed algorithm as long as feasible solutions exist.

## 6. Conclusions

A new probabilistic–geometric approach has been proposed for detection and avoidance systems for future UTM. The proposed algorithm can enable UAVs to avoid multiple moving intruders and large irregularly shaped no-fly zones, considering the detection uncertainties in sensors. The main ideas are to compute collision probability with uncertainties and to utilise it to detect a conflict and adjust the avoidance manoeuvres. This fills the gaps in research where most of the UAV collision avoidance algorithms are based on circular-shaped obstacle avoidance, and the effect of sensor uncertainties on the collision avoidance performance is largely unknown. The proposed algorithm is validated by both theoretical and numerical analysis, compared with benchmark algorithms. The result suggests that the proposed approach can provide safety, efficiency, and scalability to the future UTM solution. A future direction of this research is suggested as an extension to fully utilise cooperative UAVs’ flight plans. While the current approach is applicable to uncooperative UAVs with their position and velocity measured from the sensors, the efficiency could be improved by further utilising the flight plan and manoeuvre strategy in the case of cooperative UAVs. The research objective will be to provide an analytical safety guarantee compared with other learning-based methods [31].

## Figures and Tables

**Table 1:** Simulation parameters.

DGC	APF	PSO
$\begin{array}{cc} K\; & 1\\ R_{i,RC} & 250\;m\\ R_{j,RC} & 3r_{j} \end{array}$	$\begin{array}{cc} k_{way}\; & 1\\ k_{in} & h(R_{in,j}\left(t\right)-55)\\ k_{obs} & h(R_{i,centre}\left(t\right)-75) \end{array}$	$\begin{array}{cc} \lambda \; & 100\\ \mathrm{No}.\;\mathrm{of}\;\mathrm{particles} & 30\\ \mathrm{No}.\;\mathrm{of}\;\mathrm{iterations} & 20 \end{array}$

**Table 2:** Simulation Results.

	Safety	Efficiency	Scalability
	(Minimum Distance (m))	(Total Flight Time (s))	(Computational Time (s))
DGC	49.29–51.34	99.9–119.6	0.2
APF	8.21–104.83	114.1–154	0.1
PSO	42.77–66.98	91–159.6	4.6

## Data Availability

Not applicable.

## Abbreviations

The following abbreviations are used in this manuscript:

APF	Artificial Potential Field
ATM	Air Traffic Management
DGC	Differential Geometry Concept
PSO	Particle Swarm Optimisation
UAV	Unmanned Aerial Vehicle
UTM	Unmanned Aircraft Traffic Management
